# Human Health and Climate Change: Leverage Points for Adaptation in Urban Environments

**DOI:** 10.3390/ijerph9062134

**Published:** 2012-06-06

**Authors:** Katrina Proust, Barry Newell, Helen Brown, Anthony Capon, Chris Browne, Anthony Burton, Jane Dixon, Lisa Mu, Monica Zarafu

**Affiliations:** 1 National Centre for Epidemiology and Population, The Australian National University, Canberra, ACT 0200, Australia; 2 Fenner School for Environment and Society, Research School of Engineering, The Australian National University, Canberra, ACT 0200, Australia; Email: barry.newell@anu.edu.au; 3 School of Public Health, Curtin University, GPO Box U1987, Perth, WA 6845, Australia; Email: h.brown@curtin.edu.au; 4 Discipline of Public Health, Faculty of Health, University of Canberra, ACT 2601, Australia; Email: tony.capon@canberra.edu.au; 5 Research School of Engineering, The Australian National University, Canberra, ACT 0200, Australia; Email: chris.browne@anu.edu.au; 6 School of Medicine, University of Western Sydney, Locked Bag 1797, Penrith, NSW 2751, Australia; Email: 17111788@student.uws.edu.au; 7 National Centre for Epidemiology and Population, The Australian National University, Canberra, ACT 0200, Australia; Email: jane.dixon@anu.edu.au; 8 Public Health and Preventive Medicine Residency Program, School of Population & Public Health, University of British Columbia, Vancouver, BC V6T 1Z4, Canada; Email: lisajjmu@gmail.com; 9 University of Technology Sydney, 15 Broadway, Sydney, NSW 2000, Australia; Email: monica.e.zarafu@student.uts.edu.au

**Keywords:** cities, urban health, climate adaptation, systems thinking, system dynamics, conceptual models, co-effects, leverage points

## Abstract

The design of adaptation strategies that promote urban health and well-being in the face of climate change requires an understanding of the feedback interactions that take place between the dynamical state of a city, the health of its people, and the state of the planet. Complexity, contingency and uncertainty combine to impede the growth of such systemic understandings. In this paper we suggest that the collaborative development of conceptual models can help a group to identify potential leverage points for effective adaptation. We describe a three-step procedure that leads from the development of a high-level system template, through the selection of a problem space that contains one or more of the group’s adaptive challenges, to a specific conceptual model of a sub-system of importance to the group. This procedure is illustrated by a case study of urban dwellers’ maladaptive dependence on private motor vehicles. We conclude that a system dynamics approach, revolving around the collaborative construction of a set of conceptual models, can help communities to improve their adaptive capacity, and so better meet the challenge of maintaining, and even improving, urban health in the face of climate change.

## 1. Introduction

Since the Industrial Revolution there has been a flow of human beings from rural settings into cities. These immigrants seek employment, education, health and social services, cultural activities, and protection from adverse environmental conditions. Urban population growth has continued in recent decades, and today more than half the world’s people live in cities and towns. This trend is continuing, and the world’s urban population is predicted to reach some five billion by 2030 [1].

While many people benefit from their membership in urban communities, and find cities stimulating centres of innovation and opportunity, the negative consequences of high-consumption living are becoming apparent in some urban settings. These consequences are particularly important in relation to public health and well-being. City dwellers are confronted, among other things, with air and noise pollution, heat-island effects, reduced opportunities for physical activity and rest, and the high cost of fresh food [2,3,4]. In an increasing number of cases, people suffer from overcrowding, alienation, inequity, and high crime rates. Furthermore, when looked at more widely, some cities are seen to have large ecological footprints. Urban communities can be intense users of energy, water and other natural resources, and are prolific producers of greenhouse gases (GHG) and waste.

Traditionally such issues have been approached on a one-by-one basis. It is a natural human response, when faced with a suite of problems, to attempt to solve each one separately. Solutions are looked for locally, and connections between problems are overlooked or downplayed. For example, because urban communities are relatively isolated from the natural world, urban dwellers can be blind to their own dependence on fundamental ecosystem services—with the result that environmental issues are often seen as peripheral. Experience has shown, however, that such “silo” approaches are ineffective and misleading. Cities are complex systems. Their behaviour emerges from interactions between their parts (actors, sub-systems, sectors), and between their parts and the components of the wider Earth system. It is, therefore, not possible to understand the behaviour of a city by studying the behaviour of its parts taken separately, in isolation from one another. A “systems approach”, with a strong focus on cross-sector interactions, is needed [5].

Nowhere is the need for a systems approach more apparent than in the public health arena. There is a growing recognition today that physical, mental, and social health are closely interconnected, and strongly affected by decisions that are made in other sectors (e.g., transport, energy, agriculture, environment). Public-health policy makers now seek to develop more effective strategies that mesh insights from a wide range of perspectives. The urgency of this integrative challenge is increased by the public health risks imposed by climate change. The Intergovernmental Panel on Climate Change (IPCC) has reported that climate change is already contributing to the global burden of disease and premature death, and that these effects are likely to increase over time in all countries [6].

Climate change projections include increases in extreme events (heatwaves, droughts, floods, wildfires), and changes to environmental determinants of health such as air, food and water quality. The nature and extent of these impacts is strongly influenced by environmental factors, and so urban populations are likely to experience impacts of climate change in ways that are significantly different to those in non-urban environments [3,7,8,9,10,11,12]. Given the expected interactions between urban form, climate and public health, it is clear that the adaptation and mitigation strategies designed for urban areas must have a secure foundation in systems science. If systemic approaches to urban climate adaptation can be devised, they will have the potential to affect the health and well-being of more than half of the World’s population, and so will be crucial components of the worldwide response to climate change [3].

Nevertheless, practical attempts to take a systems approach soon run into a major problem. An urban climate-health system is overwhelmingly complicated. There are many components interacting with each other in many different, time-dependent ways. There is uncertainty concerning the nature of a significant number of these interactions, and ignorance of the existence of others. Efforts to construct complete models of such systems are unlikely to generate useful results—such models involve too many variables and too much uncertainty to provide clear guidance. What is needed is a way to cut through the complexity—a way to identify the essential drivers of system behaviour. Research in System Dynamics [13] has demonstrated that this is possible. That is, there is a way to reconcile (a) the need to take a broad systems view, with (b) the need to produce policy recommendations that are specific enough to be of practical value. In many circumstances it is possible to isolate relatively simple, generic feedback structures that can explain significant aspects of the behaviour of a wide range of real-world systems. Discussions centred on a small set of such “conceptual models” can yield useful insights into the dynamics of a given system-of-interest, and so represent a valuable first step in attempts to improve management effectiveness and adaptive capacity [5,13,14].

Our aim in this paper is to demonstrate how such conceptual models can be constructed. A basic understanding of the dynamics of complex systems is a necessary point-of-departure for our discussion. For this reason, we begin, in Section 2, by outlining some key concepts from the field of System Dynamics [15]. We summarise the idea of “leverage points”—places in a system where relatively small management intervention can produce large changes—and briefly introduce the *Collaborative Conceptual Modelling* (CCM) approach that underlies the model-building process described here. CCM includes a three-step process that has the potential to enhance a group’s adaptive capacity:

(1) *System Template*. Develop an abstract “system template” that expresses a generic hypothesis concerning the behaviour of human-environment systems. The system template described in Section 3 is a “co-effects” model that was developed in an Australian project focused on the nexus between urbanism, climate adaptation, and human health.
(2) *Problem Space*. Develop an intermediate-level version of the template that projects it into the domain of the type of management problem of concern to the group. The model presented in Section 4 is tailored to support a discussion of the impacts of “maladaptive dependence” in urban systems. This choice of problem space is based on our focus on the growth of “maladaptive dependencies” as a powerful force in urban climate-health systems.
(3) *System-of-Interest*. Generate a low-level version of the template that is tightly focused on a particular system-of-interest. This step requires the identification of (a) specific system variables, and (b) the specific interactions that can take place between these variables. The model presented in Section 5 is focused on selected maladaptive aspects of urban dependence on private motor vehicles (cars). It is a version of the intermediate-level template, instantiated to fit the interactions between urban transport, local air quality, and cardiovascular and respiratory disease.

In Section 6 we apply the ideas developed in the previous sections to a discussion of leverage points for adaptation in the urban climate-health system. Concluding remarks are given in Section 7.

## 2. Elements of a System Dynamics Approach

The word “system” has many meanings. In this paper we are concerned with system dynamics. By “dynamics” we mean the way that the state of a system changes over time in response to endogenous (internally generated) or exogenous (externally imposed) forces. We define a *dynamical* system to be:

… something composed of discernible parts (elements, agents) that interact to constrain each others’ behaviour. It is these *mutual constraints*, operating between the parts of the system, that limit the range of behaviours available to the system as a whole, and thus give rise to its “emergent” (or synergistic) properties [16].

**Figure 1:**
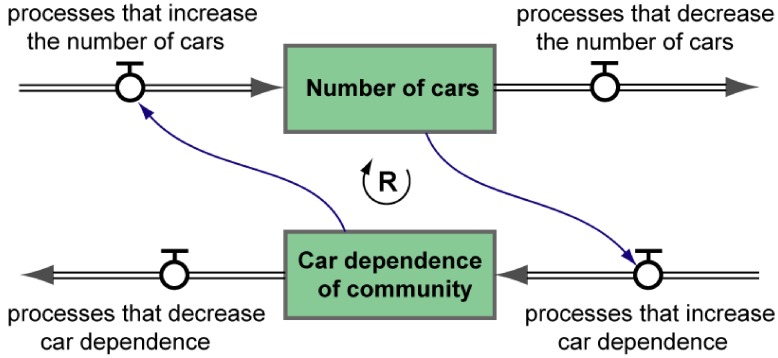
An illustrative stock-and-flow feedback structure. The rectangles represent stocks (accumulations). The double-lined arrows represent inflows and outflows (processes that change the amounts accumulated). The “tap” symbols represent process flow-rates. The single-lined arrows represent influence or information links. The encircled R in the centre of the diagram indicates that this is a reinforcing (positive) feedback loop.

The concept of system “behaviour” can be understood using the Bathtub Metaphor, and the related stock-and-flow language, developed by the System Dynamics community [5,13,14,17]. In the Bathtub Metaphor the volume of water in the tub represents the “stock” of material or non-material things accumulated in a system, and the “flows” of water into and out of the tub represent the effects of processes that change the “level” of the stocks. The way that these levels change over time is referred to as “the behaviour over time” of the system. It is important to recognise that the Bathtub Metaphor is not just a matter of colourful language—it allows strict “bathtub logic” to be used to think about system behaviour in general [18]. That is, just as the water level in the bathtub changes over time in a way that depends on changes in the *difference* between inflow and outflow rates, so the state of a system changes over time in a way that depends on changes in the relative rates of its state-change processes [19]. A readable introduction to System Dynamics is given by Meadows [5], and the field is thoroughly reviewed by Sterman [13]. A System Dynamics approach revolves around four crucial concepts:

(1) *Stocks*—Stocks are accumulations of material and non-material things. For example, a store’s inventory of products for sale, the number of vehicles in a city, urban population, the number of people with a given disease, the power of Local Government. Accumulation occurs whenever a “container” integrates the difference between its inflows and outflows over time. “Accumulation is a pervasive process in everyday life, and arises at every temporal, spatial and organisational scale” [20]. The state of a system at any given time can be described by reporting the current levels of its stocks (*i.e.*, the amounts accumulated). In other words, a system’s stocks are its “state variables”. Containers cannot be filled or drained instantaneously, so stocks cause delays in a system’s response to management initiatives. They give a system the equivalent of physical inertia and can cause oscillations in system state. Stocks also act as “buffers” between unequal inflows and outflows—for example, a city’s water-storage dam acts as a buffer between irregular river flows and steady consumption by the city dwellers. Stocks are conventionally represented by rectangles in diagrams of the structure of dynamical systems (Figure 1).
(2) *Flows*—Flows are processes that change the levels of the stocks in a system. Given that a system’s stocks are its state variables, flows are properly called “state-change processes”. Inflows increase the level of a stock, outflows reduce its level. The level of a stock can change only if there is a net inflow or outflow. The rate at which the state of a system changes depends on the process flow rates (represented by the “tap” symbols in Figure 1). The stock-and-flow structure of a system determines the general form of its behaviour over time. Stocks cannot affect each other directly—they communicate via flows. A change in the level of a stock can affect the rate of a flow that, in turn, affects the level of another stock. A clear distinction between stocks (accumulations) and flows (processes) is a hallmark of good systems thinking.
(3) *Feedback*—Dynamical systems contain causal loops. A change in the level of a stock can feed back, around a causal loop, to either amplify or oppose the original change. A feedback structure that amplifies change is called a “reinforcing” (positive) feedback loop. A feedback structure that opposes change is called a “balancing” (negative) feedback loop. A simple reinforcing feedback structure is shown in Figure 1, where an increase in the car dependence of the community increases the rate of growth of the car fleet. As the car fleet grows so private and public decisions are made that increase car dependence in the community, and so on around the loop. Such a loop can also drive the level of both stocks down, reducing both the car dependence of the community and the size of the car fleet.
(4) *Endogenous Behaviour*—A distinguishing characteristic of System Dynamics thinking is its focus on endogenous behaviour [21]. Such behaviour is generated by feedback within the dynamical system—it does not need an exogenous driver. When external forces are applied, the system’s response is generated by its internal dynamics. For example, the simple reinforcing feedback structure shown in Figure 1 is capable of autonomously driving both stocks up or down. If an external force acts (say) to increase the rate of the process that decreases the car dependence of the community, then the processes that increase the number of cars will slow and the size of the car fleet will decrease over time (all else being equal). The reinforcing loop will act to amplify that change. This endogenous feedback process can continue to drive system behaviour, even after the external force has been removed.

The identification of leverage points for management intervention in a system is a principal aim of System Dynamics studies. A leverage point is a place in a system where (a) a relatively small local change can produce major effects throughout the system, and (b) communities are likely to be willing, and able, to make the required change. Meadows [5] provides an overview discussion of the nature of leverage points. She includes a useful classification of kinds of leverage points, expressed in system dynamics terms and ordered according to effectiveness. The following list of system leverage-points, in order of *increasing* power, is adapted from Meadows’s Chapter 6:

- *Numbers*. Constants and parameters such as subsidies, taxes, and standards.
- *Buffers*. The size of stabilising stocks and inventories relative to their flows.
- *Stock-and-Flow Structures*. Physical systems and the way that they interact.
- *Delays*. The length of time delays relative to the rates of system change.
- *Balancing Feedback Loops*. The strength of stabilising loops relative to the changes that they oppose.
- *Reinforcing Feedback Loops*. The strength (gain) of the driving loops.
- *Information Flows*. The structure of who does and does not have access to information.
- *Rules*. Policies and laws, including incentives, punishments, and constraints.
- *Self-Organisation*. The ability of the system to change its own structure.
- *Goals*. The purpose or function of the system.
- *Paradigms*. The mind-set out of which the system arises. This mind-set determines the system’s goals, structures, rules, delays, and parameters.

Policy makers and managers do not have to be expert system analysts, but they do need to be systems thinkers. Dynamical systems act in ways that can surprise conventional thinkers [5,13,22,23,24]. Many people have a “linear” view of the way that cause and effect operate in their world. If I push twice as hard on the pedals, the bike will go twice as fast. If two cows lean on a fence, their combined force will be simply the sum of their individual forces. Dynamical systems do not always work this way. A small applied force can lead to runaway behaviour, as a reinforcing feedback loop takes hold and amplifies the original effect. Or a large applied force can have little effect, if it is opposed by powerful balancing feedbacks. Decision makers who are linear thinkers, and focus exclusively on sectors or sub-systems small enough to appear understandable and manageable, are likely to be surprised by counterintuitive policy outcomes. Policies that emerge from a narrowly focused “silo” approach may work initially. They are, however, often ineffective (even damaging) in the medium-to-long term, because of their authors’ failure to take account of the non-linear effects of accumulation and cross-sector feedback.

Newell and Proust [25,26] have developed an approach, called *Collaborative Conceptual Modelling* (CCM), which is designed to support an integrative group’s efforts to improve their understanding of the basic dynamics of their system-of-interest, thereby improving their adaptive capacity. CCM draws on insights from three intellectual domains: cognitive science, dynamical systems theory, and the practice of applied history. It provides ways to elicit individuals’ worldviews, and to compare and mesh them to produce shared understandings that are more powerful than those that can be developed by individuals working alone [27]. It uses a set of protocols for developing “dynamic hypotheses” that describe simple feedback structures that have the potential to dominate system behaviour.

From the research point-of-view, the CCM approach provides a way to test the hypothesis that profound improvements in adaptive plans can flow from collaborative attempts to construct simple conceptual models. A CCM team works to develop a set of simple causal structures that they believe capture important aspects of the feedback dynamics of their system-of-interest. Under the CCM hypothesis, discussions of the potential interactions between these separate structures provide a way for the team to build an improved, *shared* understanding of the behaviour of dynamical systems in general, and their system-of-interest in particular. In the process, team members make the transition from “linear thinking” to “systems thinking”. Furthermore, their conceptual models are ideal starting points for the development of working stock-and-flow models that can support the development of systemic policies. The collaborative process, focused as it is around the development of cross-sector models, can also result in improved dialogue and trust between players who initially hold conflicting worldviews. For all of these reasons, the collaborative development of conceptual models has the potential to lead to increased adaptive capacity and more effective adaptive plans. The way that the CCM process can work, and the beneficial effects that systems thinking can have on adaptive planning, are discussed in the following sections.

## 3. System Template—Co-Effects in Urban Systems

The first CCM step calls for the development of an abstract “system template” that expresses a generic hypothesis about the behaviour of human-environment systems. The template presented in this paper is focused on the interplay between urban form, the state of the planet, and human health and well-being.

Some 75 per cent of Australians now live in cities of more than 100,000 people, located largely in the narrow coastal zone [28]. The interconnections between urbanism, climate adaptation, and public health have been targeted in a recent initiative of the Australian Commonwealth Scientific and Industrial Research Organization (CSIRO). CSIRO has funded a cluster of research projects (hereinafter referred to as the Cluster) under its Climate Adaptation Flagship. A principal aim of the Cluster research is to develop insights that can help urban planners and policy makers to improve their understanding of the dynamics of urban climate-health systems, thereby increasing the likelihood that their climate adaptation strategies will be effective. The Cluster projects include three focused on vector-borne diseases in Northern Australia, and three focused on the rapidly growing outer area of Western Sydney.

Sydney, the capital of New South Wales, is the largest Australian city with a population of over 4.3 million people. Constrained by the sea to the east, Sydney’s metropolitan area has been expanding westward in recent decades. The Western Sydney projects are concerned with (a) thermal stress, built environments and health, (b) urban food systems, climate change and health, and (c) urban transport, air quality, climate change and health. It is anticipated that the findings from these projects will be relevant to the outer metropolitan areas of cities elsewhere in Australia, North America and New Zealand. Cities in these countries share similar patterns of development, which reflect particularly the industrial advances of the nineteenth and twentieth centuries.

The Cluster includes a seventh project which aims to develop conceptual models and adaptation scenarios that incorporate the results from the three Western Sydney projects. Here we present the results of a series of CCM workshops that were carried out as a part of this integrative project. The aim of these workshops was to use insights from the individual projects inductively to develop an overarching system template. The process began with three project-focused workshops, followed by an integrative workshop where members of the project teams came together to develop their initial high-level view of the urban climate-health system. 

Participants in the initial, project-focused workshops were introduced to basic system dynamics concepts and were instructed in the use of influence and causal-loop diagrams as exploratory system-analysis tools. Applying the CCM “pair-blending” approach [27], participants worked (a) individually to produce influence diagrams that expressed their particular view of the causal structure of their system-of-interest, and then (b) in pairs to develop “blended” influence diagrams that meshed their individual views. This step helped them develop a new, shared view of the target causal structure. Finally, the pair-blended diagrams were presented and discussed in plenary session.

**Figure 2:**
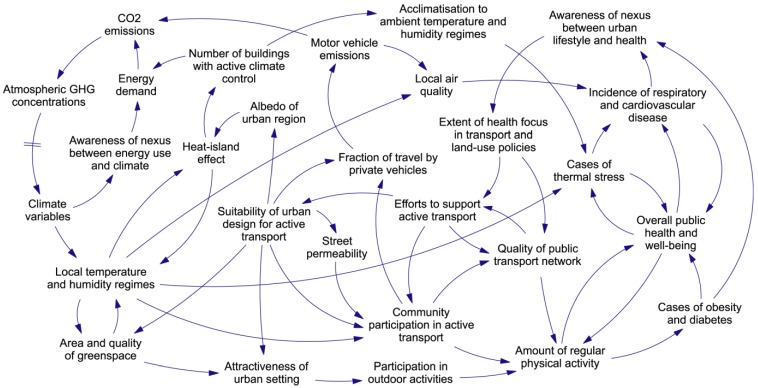
An hypothesis concerning selected aspects of the causal structure of urban climate-health systems. In this influence diagram the blocks of text represent system variables, and the arrows represent causal links.

The outputs from the initial workshops were used to construct a number of system-overview diagrams. Each diagram expressed a hypothesis regarding some of the cross-sector feedback structures in a typical urban climate-health system. An example, with a focus on the interplay between climate, transport, urban form, and public health, is reproduced as Figure 2. Diagrams of this type provide a glimpse of the feedback structure of the target system, but they are obviously too complicated to provide *useable* insights into the dynamics of the system. It is here that the CCM three-step modelling process is needed—starting with the development of the system template.

**Figure 3:**
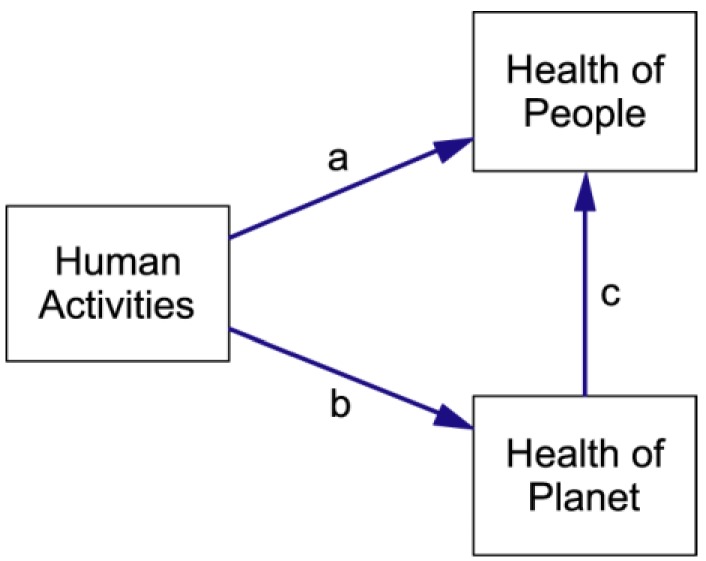
Boyden’s Biosensitivity Triangle (modified to reflect our focus on human activities). The arrows have been labelled for ease of reference.

The aim of the integrative workshop was to develop a first version of the system template. The process was guided by the diagrams produced in the initial workshops and included a consideration of Boyden’s Biosensitivity Triangle (Figure 3). Boyden [29] was concerned with the impact of human society on the health of people and the planet. He identified two pathways by which human society can affect public health—a direct pathway (shown by arrow **a** in Figure 3) and an indirect pathway (shown by arrows **b** and **c**) whereby impacts on the health of the planet flow through to changes in public health. These ideas provided a useful staring point for the integrative discussions.

**Figure 4:**
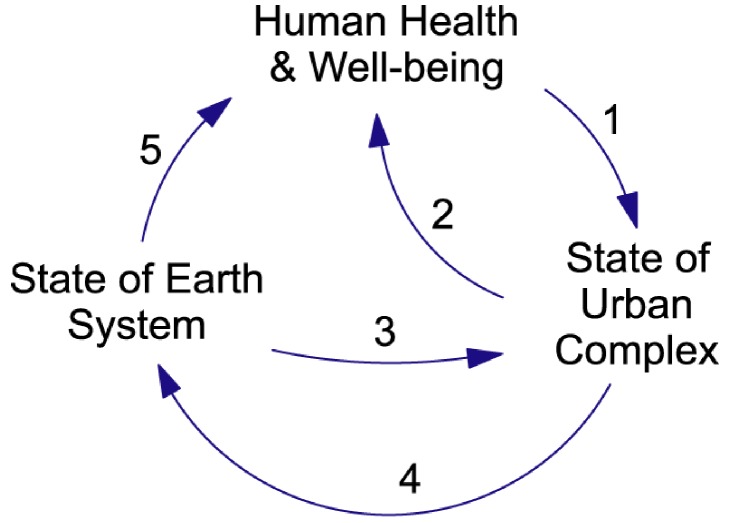
The Co-Effects Template. The blocks of text in this influence diagram represent system stocks (state variables) grouped into three high-level sub-systems. Examples of these stocks are given in Table 1. The arrows represent bundles of causal links. Examples of the flows (state-change processes) associated with each link are given in Table 2.

A system template based on the outcomes of the integrative workshop is shown in Figure 4. The template expresses our hypothesis concerning the high-level structure of urban climate-health systems. A principal aim of the Cluster research is to generate insights into the interactions between urbanism, climate and health. Accordingly, the high-level structure expressed by the template provides an explicit subdivision of system stocks into three sub-sets: **State of Urban Complex, State of Earth System** (which includes the climate sub-system and local environmental sub-systems), and **Human Health & Well-Being**. This process serves to sub-divide the overall urban climate-health system into sub-systems that are themselves complex feedback systems. Examples of the kind of stocks allocated to the sub-systems are listed in Table 1. Each arrow in the diagram represents a bundle of causal links. The kind of causal processes included in each bundle are identified in Table 2.

The structure displayed in Figure 4 is influenced by Boyden’s triangle, but differs from it in two important respects: First, the notion of **Human Activities** has been replaced with **State of Urban Complex**. This has been done to emphasise that all the variables of this sub-system are stocks (state variables). Activities are not stocks—they are flows that are affected by, and affect, the levels of the stocks. In Figure 4 human activities are represented by Links 1 to 4. Second, there are two additional causal links. Links 1 and 3 are “policy” links whereby the state of human health and well-being, and the state of the planet, drive human activities that influence the state of the urban complex.

**Table 1:** The stocks of Figure 4.

Stocks	Description
State of Urban Complex	Stocks that define the state of a city and its inhabitants. Both physical and social variables are required. Examples include area of city, area of green space, kilometres of roads, size of car fleet, quality of infrastructure, extent of infrastructure, street permeability, energy consumption, albedo of urban region, size of population, population density, security of food supply, affluence, social cohesion, alienation, equality and visual amenity.
State of Earth System	Stocks that define the physical and ecological state of the planet. Must include variables that measure the physical state of the planet and those measuring the health of ecosystems at all scales from local to global. Examples include atmospheric energy content, GHG concentrations, ocean acidity, biodiversity, species abundance, extent of native vegetation, condition of soils, and condition of fresh water.
Human Health & Well-being	Stocks that define the physiological, psychological and social health of an urban community. Examples include incidence of specific diseases, extent of obesity, physical fitness, stress levels, level of mental health, acclimatisation to weather extremes, sense of purpose, sense of belonging, sense of security.

The template has three feedback loops. There is a health-effects loop that operates through Links 1 and 2. There is an environmental-effects loop that operates through Links 3 and 4. And there is a “co-effects” loop that operates through Links 1, 4 and 5. Link 5 represents processes whereby the state of the planet has a direct effect on human health and well-being. For example, there are “co-benefits” where actions taken to mitigate climate change have significant public health benefits [30]. There are also “co-costs” where actions taken to mitigate global change have detrimental effects on public health and well-being—for example, increased environmental flows can threaten the viability of irrigation communities. The template must be able to accommodate both kinds of feedback effect—the name “Co-*Effects* Template” has been chosen to reflect the required generality.

**Table 2:** The causal links of Figure 4.

Link	Processes represented by the link
1	Human activities. The design and implementation of formal and informal social and public health policies.
2	Human behavioural patterns influenced by the state of the urban complex. Processes whereby the state of the urban complex directly affects individual physiological, psychological and social functioning.
3	Human activities. The design and implementation of formal and informal environmental protection policies.
4	Extraction of natural resources and pollution (dumping of wastes). Conservation and restoration activities.
5	Processes whereby environmental conditions directly affect human physiological, psychological and social states.

The absence of a sixth link, running directly from **Human Health & Well-Being** to **State of Earth System**, expresses our view that under normal circumstances there are no processes whereby the health and well-being of a community has a significant *direct* effect on the state of the planet. This causal link exists, but in the overwhelming majority of cases it is mediated by human activities that affect **State of Urban Complex**. That is, it operates through Links 1 and 4.

The Co-Effects Template is a high-level, abstract representation. It summarises real-world feedback structures that have literally thousands of variables and many more causal links. The use of the template to guide studies of the urbanism-climate-health nexus should help analysts to establish and maintain a systems focus. In particular, its use should ensure that cross-sectoral feedback effects are taken into account. But, to use it to guide a dynamical study, the template must be instantiated to produce a model that is tailored to the context and scales of a specific, concrete study. In CCM practice this process involves two more steps.

## 4. The Problem Space—Maladaptive Technology Dependence

The second CCM step in the development of a useful conceptual model involves projecting the system template into the domain of the chosen type of adaptation problem. In this paper our chosen problem space is the well-known tendency of urban communities to develop strong, often maladaptive, dependence on specific technologies [31,32,33]. Examples include air conditioning, private motor vehicles, fossil-fuel-based electricity generation, flood levees, television and pharmaceuticals. We consider a technology dependence to be maladaptive if, despite having definite benefits, it increases the risk that an urban community will suffer ill effects from climate change (or other global change).

**Figure 5:**
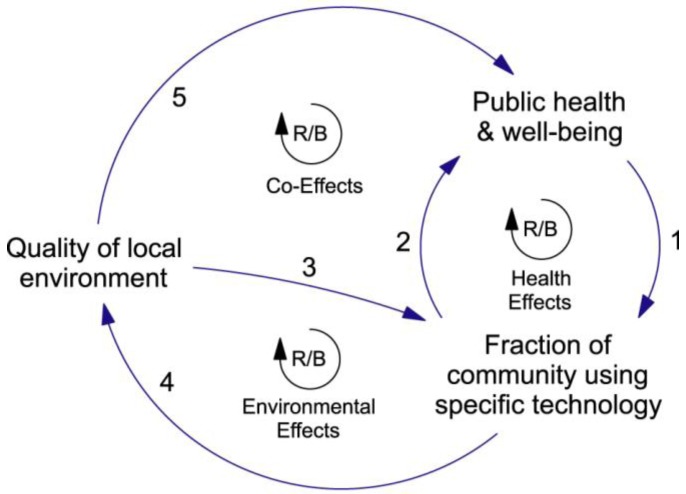
An intermediate-level hypothesis concerning technology dependence in urban settings. The structure is derived from the Co-Effects Template and the arrows (causal links) are labelled in accordance with the numbering scheme used in Figure 4. The state-change processes represented by each arrow are described briefly in Table 3. The encircled symbol (R/B) in the centre of each feedback loop indicates that the loop is either reinforcing or balancing, depending on the net effect of its causal links.

Our intermediate-level version of the Co-Effects Template is shown in Figure 5. The high-level group of stocks **State of Urban Complex** has been replaced with the single stock **Fraction of community using specific technology**, which serves to establish a focus on the issue of technology dependence. **State of Earth System** has been replaced with a more focused group of stocks labelled **Quality of local environment**, and **Human Health & Well-Being** has been replaced with **Public health & well-being**. The feedback loops in Figure 5 have been labelled Health Effects (Links 1 and 2), Environmental Effects (Links 3 and 4), and Co-Effects (Links 1, 4 and 5). The causal processes that are associated with each link are described in Table 3.

**Table 3:** The causal links of Figure 5.

Link	Processes represented by the link
1	Design and implementation of social and public health policies that target the community’s dependence on the specific technology.
2	Processes whereby the habitual use of the specific technology affects the state of human physiological, psychological and social systems. There will typically be a mixture of positive and negative effects.
3	Design and implementation of environmental policies that target the community’s level of dependence on the specific technology.
4	Processes whereby the habitual use of the specific technology affects the state of the local environment. These processes are mediated by local ecosystems through the provision of ecosystem services. Extraction of resources and dumping of wastes. Conservation and restoration processes. Processes typically have a mixture of positive and negative effects.
5	Processes whereby environmental conditions affect the state of human physiological, psychological and social systems.

Each link in Figure 5 represents a number of causal processes—the *net effect* of these processes depends on whether they reinforce or oppose each other. At this intermediate level the stocks and causal links shown in the diagram are still too abstract to allow dynamical analysis, and so the nature of the feedback loops (whether they are balancing or reinforcing) cannot yet be determined. Nevertheless, the diagram can be used to develop a general classification of adaptation situations.

Such a classification is shown in Table 4. In the first column of this table are listed type-designations for adaptation situations where there is a risk of maladaptation. In columns two and three are listed the polarities of Links 2 and 4, respectively (see Box 1). Column four of Table 4 lists the risk of maladaptation. In column five, we list policy actions that have the potential to promote adaptation.

**Table 4:** Classification of adaptation situations.

Type	Link 2 Polarity	Link 4 Polarity	Level of risk	Potential adaptive strategy
**I**	Positive	Positive	Zero	Increase Dependence
**II**	Negative	Negative	High	Decrease Dependence
**III**	Positive	Negative	Medium	Decrease Link 4 Effect
**IV**	Negative	Positive	Medium	Decrease Link 2 Effect

**BOX 1 Causal Link Polarities**

In System Dynamics terminology a causal link can have one of two polarities [13]. In the diagram below, the letters **A** and **B** represent system variables and the arrows represent causal links. The “polarity” of a link is indicated by a plus sign (+) or a minus sign (−) attached to the arrow representing the link.

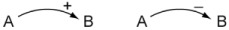

Positive polarity means that an increase/*decrease* in the level of variable **A** will cause the level of variable **B** to eventually rise above/*fall below* the level that it otherwise would have had (all else being equal). Similarly, negative polarity means that an increase/*decrease* in the level of variable **A** will cause the level of variable **B** to eventually fall below/*rise above* the level that it otherwise would have had (all else being equal). Diagrams where polarities have been assigned are called causal diagrams or causal-loop diagrams.

When Links 2 and 4 both have positive polarity, their associated processes work together to *improve* the health of the environment and the community, and the risk of maladaptation will be zero (all else being equal). In such Type I adaptation situations, technology dependence produces net positive outcomes and adaptation may well be enhanced by increasing the community’s use of the specific technology. When Links 2 and 4 both have negative polarity, their associated processes work together to *reduce* the health of the environment and the community, and so the risk of maladaptation will be high. In such Type II adaptation situations, characteristic of maladaptation, it will be advantageous in the long term to reduce the community’s use of the technology. When the net polarities are mixed (Type III and IV leverage points), then the Links 2 and 4 processes will oppose each other and the risk of maladaptation will be reduced. In Type III situations, where Link 2 has net positive effects and Link 4 has net negative effects, it may be useful to maintain the current level of community dependence on the technology, but to work to reduce the impact of Link 4 processes. Finally, in Type IV situations, it may be effective to maintain the level of dependence but seek ways to reduce the impact of the Link 2 processes. Note that, in this analysis, we assume that an increase in the quality of the local environment always has a beneficial effect on public health and well-being.

The classification presented in Table 4 must be used with caution. First, because feedback systems are non-linear, the net polarity of a bundle of causal links can change as the state of the system changes, causing a shift from beneficial to detrimental impacts. Second, because the feedback system shown in Figure 5 is clearly a sub-system of the wider human-environment system, in some cases the impact of changes in the wider system may invalidate the reasoning behind the classification. Nevertheless, the classification typifies the kind of reasoning that is involved in systems thinking and, provided that it is used with understanding, it can provide a useful starting point for dynamical analysis.

## 5. System-of-Interest—Dependence on Private Motor Vehicles

The third CCM step in the development of a useful conceptual model involves defining a particular system-of-interest. This instantiation process involves the following steps:

(1) Replace the groups of stocks with the specific stocks of concern in the particular dynamical study. The number of stocks included in the model needs to be small (ideally ~5). Maintain the distinction between stocks (state variables) and flows (state-change processes).
(2) Describe the specific flows (state-change processes) whereby the chosen stocks affect each other. There will be a mixture of beneficial and detrimental effects.

To keep our model development focused on the co-effects of technology dependence, we follow the dynamic hypothesis displayed in Figure 5. For this illustrative case study we have chosen private motor vehicles powered by internal combustion engines as the technology of interest. The benefits and costs of dependence on this technology are widely recognised by urban designers and managers [34,35,36].

**Figure 6:**
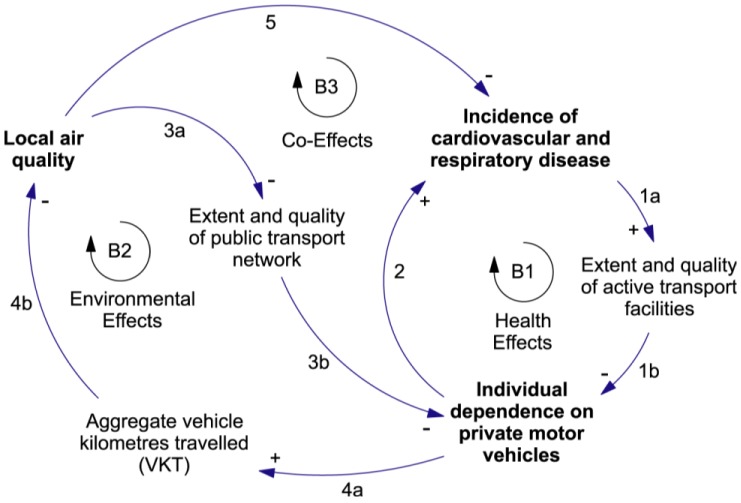
A causal loop diagram concerning selected co-effects of vehicle-dependence in urban settings. The arrows represent causal links and are labelled in accordance with the numbering scheme used in the Co-Effects Template. Each arrow has been assigned a polarity. The state-change processes represented by each arrow are described in Table 5. Links 1, 3 and 4 each have two components, labelled “a” and “b”. The encircled symbols B1, B2 and B3 indicate that all three feedback loops are balancing.

As shown in Figure 6, we have selected **Individual dependence on private motor vehicles** as our indicator of technology dependence. This stock can be measured, for example, in terms of the fraction of an individual’s travel that is made using cars. **Local air quality** has been selected as the measure of the quality of the local environment. In addition to the direct effect of vehicle emissions, this stock is affected by climatic conditions—examples include the dependence of ground-level ozone concentrations on sunlight and temperature regimes, and the dependence of local air quality on the flushing effect of wind. **Incidence of cardiovascular and respiratory disease** is our measure of public health and well-being. This stock is actually an indicator of public ill-health, but it is used here because measures of the incidence of disease are a natural choice in the public health arena.

Intervening stocks have been added in Links 1, 3 and 4 to clarify the logic of our hypothesis. **Extent and quality of active transport facilities** (Link 1) and **Extent and quality of public transport network** (Link 3) are intended to represent the result of policies designed to influence an individual’s chosen mode of transport. **Aggregate vehicle kilometres travelled** (Link 4) is the sum of the distance travelled by all members of the community. All three of these intervening stocks effect a scale-change, from regional scale to individual scale (in the case of Links 1 and 3) and from individual scale back to regional scale (in the case of Link 4). The processes whereby the selected stocks affect one another are described briefly in Table 5.

Once specific stocks have been chosen it becomes possible to assign polarities to the causal links. The polarities assigned in Figure 6 indicate that an increase in the fraction of travel by cars will lead to a decrease in local air quality and an increase in the incidence of disease—a Type II adaptation situation (Table 4). The obvious adaptive strategy is to implement health and environmental policies that work to reduce the community’s dependence on cars. This conclusion is given additional weight by the observation that cars powered by internal combustion engines contribute significantly to atmospheric GHG concentrations and climate change.

It is important to recognise that balancing feedback loops like those shown in Figure 6 are “goal-seeking” [13]. That is, all else being equal, the feedback will drive the level of the controlled stock towards a specific target level. In situations where the controlled stock is already at the target level, a balancing loop will resist any force that tends to move it away from the target level.

**Figure 7:**
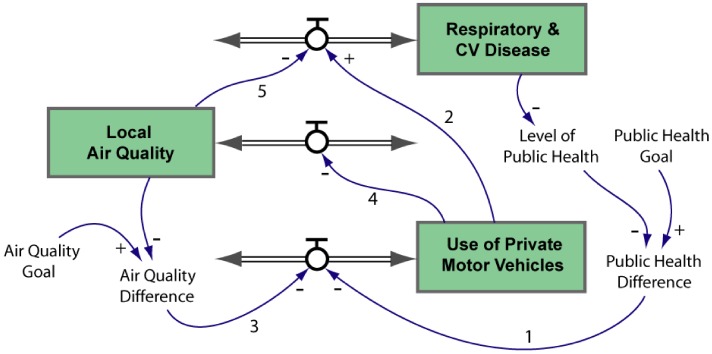
A stock-and-flow version of the co-effects structure of Figure 6. The graphical conventions are those defined for Figure 1. The double-lined, double-ended arrows are “bi-flows” representing net processes that can drive the levels of their affected stocks up (inflow) or down (outflow). The variables labelled **Public Health Difference** and **Air Quality Difference** represent the difference between the actual level of the controlled variables and their respective goals.

A stock-and-flow map, such as that displayed in Figure 7, can help explain this behaviour. Consider, for example, the Environmental Effects loop (labelled B2 in Figure 6). The strength of the feedback exerted by this loop depends on 

                                 **Air Quality Difference = Air Quality Goal−Local Air Quality.**

As the level of **Local Air Quality** approaches **Air Quality Goal**, **Air Quality Difference** will approach zero, as will the strength of the balancing feedback. As a result, **Local Air Quality** will stabilise at or near the goal level. In many real-world systems the controlled stock will follow a damped oscillation about its corresponding goal. Oscillatory behaviour is the result of the delays caused by the filling and draining of stocks. Because of these delays the controlled variable cannot respond instantly to the difference between the actual and desired conditions and so overshoots and undershoots its goal. In general terms, however, the overall level of **Local Air Quality** can be expected to rise if **Air Quality Goal** is lifted. The same behaviour will be displayed by the Health Effects loop (B1) and the Co-Effects Loop (B3). Note that the latter is driven by the public health goal.

**Table 5:** The causal links of Figure 6.

Link	Processes represented by the link
1a	Design and implementation of policies that promote active transport, driven by the insight that cardiovascular and respiratory problems can be ameliorated by physical activity. Processes include communication of research results and political processes. Political will is required. Policy initiatives include allocation of public funds for construction and maintenance of active transport facilities (e.g., bike paths), and allocation of private funds for the purchase of equipment for active transport (e.g., bikes).
1b	Individual decisions to use active transport instead of vehicular travel. Factors that influence the attractiveness of active transport for commuters include: commuting distance, individual physical fitness, quality and extent of available facilities (including work-place showers), air quality, and weather. Processes include public education.
2	Obesogenic effects of reduced physical activity, particularly for commuters. Immune system suppression due to chronic psycho-social stress.
3a	Design and implementation of policies that promote public transport. Processes include communication of research results and political processes. Political will is required. Policy initiatives include allocation of public funds for expansion and maintenance of the public transport system.
3b	Individual decisions to use public transport instead of vehicular travel. For commuters the attractiveness of public transport flows from reductions in the cost and stress of commuting, but depends also on the number and location of pick-up points, and the facilities provided at those points. Processes include public education.
4a	The process of driving cars.
4b	The process of using vehicle propulsion systems. Internal combustion engines emit a range of air pollutants including CO, CO_2_, NO_x_, VOC and fine particulate matter. In the presence of sunlight NO_x_ and VOC contribute to the formation of ground-level O_3_. Effect on air quality mediated by local ecosystem through the provision of ecosystem services.
5	Reduction in the capacity of haemoglobin to transfer oxygen. The induction of inflammatory responses. Disrupted cellular processes. Increased oxidative stress. Immune system impairment.

**Figure 8:**
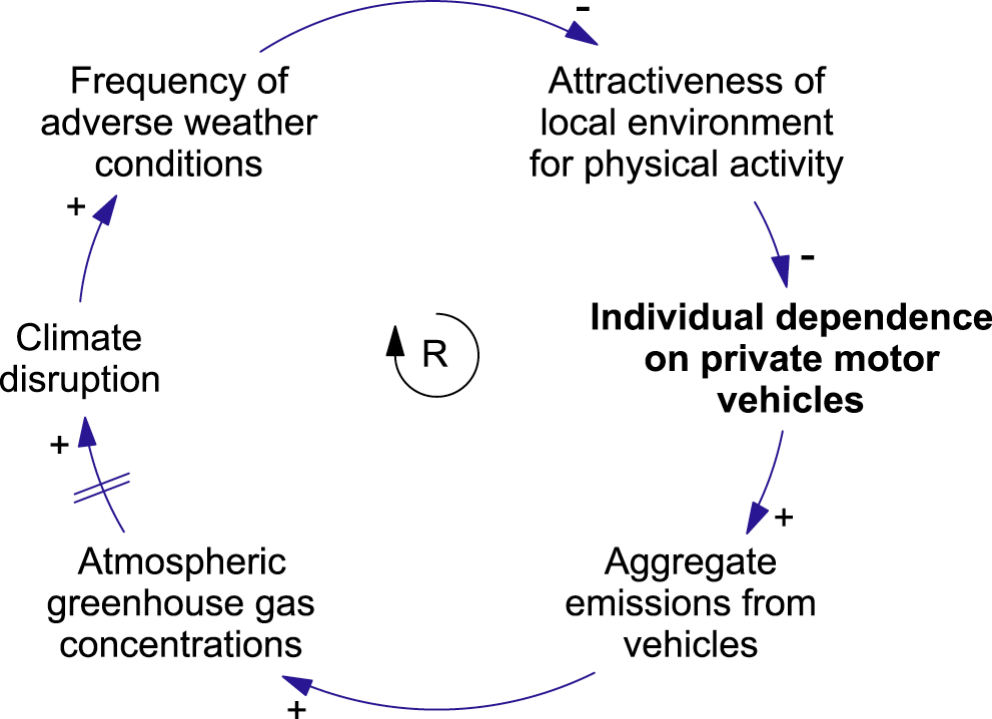
A dynamic hypothesis concerning the interaction between car dependence and climate change. The short parallel lines crossing the link from **Atmospheric greenhouse gas concentrations** to **Climate disruption** indicate a delayed effect.

The co-effects feedback structure presented in Figure 6 and Figure 7 is tightly focused on a specific group of variables. A more global view is presented in Figure 8. The cross-scale reinforcing loop shown in this figure can drive a growing dependence on cars as the climate becomes less and less suitable for outdoor activity. It shows how local behaviour can affect planetary health in the long term. While individuals may not consider that their actions matter at the scale of the climate system, the aggregate effect of a growing worldwide dependence on cars is likely to be a significant contributor to climate change [37].

The feedback structures shown in Figure 6, Figure 7, Figure 8 illustrate why car dependence is maladaptive, at least from the point of view of those concerned with long-term trends in human and planetary health. Nevertheless, these considerations are not of immediate concern to many urban dwellers. Cars provide a level of flexibility, independence, and social status that is highly valued. This, in large measure, explains why such a strong dependence on the technology developed in the first place, and why it continues to grow worldwide. The adaptive challenge is to identify urban leverage points that are powerful enough to reverse this trend. And this requires an appreciation of the endogenous forces that drive the dependence.

## 6. Discussion: Urban Leverage Points and Adaptive Capacity

**Figure 9:**
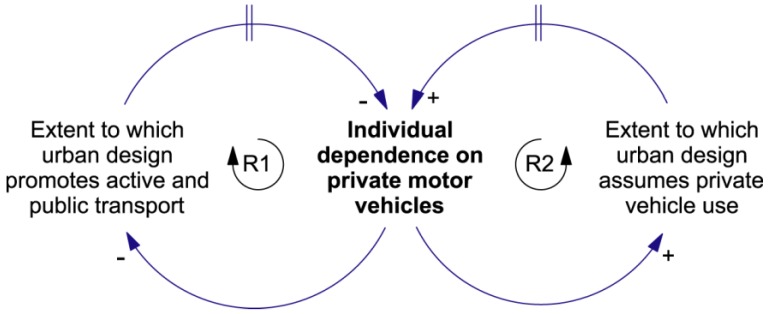
The Adaptive Challenge. This causal loop diagram instantiates the feedback structure of the Success to the Successful archetype. There are two reinforcing loops, R1 and R2, that work together to affect the extent to which individuals depend on cars. This feedback system has contributed to the growth of car dependence in modern cities. The challenge is to reverse this trend.

The co-effects structure shown in Figure 6 and Figure 7 captures one side of the urban adaptation story, namely the potential stabilising force of policies designed to counteract growing car dependence. Another side of the story involves the reinforcing feedback structures that currently act to increase car dependence. Figure 8 and Figure 9 (below) provide examples. While it may seem counterintuitive, these growth structures also have the potential to reduce car dependence. The balancing loops of Figure 6 work to oppose changes in a community’s level of dependence. Reinforcing feedback loops are more versatile. They can amplify changes in dependence—either up or down. Such destabilising structures are therefore potentially powerful leverage points. Here, as an example, we discuss the reinforcing interactions that exist between car use and urban design. 

Throughout the twentieth century cars have been a significant force in the evolution of cities [38,39,40,41]. As cars became more affordable, the pattern of urban development reflected their impact on urban lifestyles. Many families preferred to move away from the old inner city to free-standing houses in new suburbs. Private cars made this migration possible. Then, as the city expanded beyond the reach of established transport routes, cars became a necessity. They also played an enabling role in the development of the large centralised shopping plazas that replaced the neighbourhood-scale shopping precincts of the more densely organised inner city. Urban sprawl and car dependence fed off each other in a reinforcing feedback loop [42,43].

In Figure 9 we present a causal loop diagram that summarises key interactions between car use and urban design. The reinforcing feedback structure shown in this diagram is an example of the Success to the Successful system archetype. System archetypes are simple, generic feedback structures that have been found to occur in a wide range of contexts [5,14,44]. The Success to the Successful archetype captures the dynamics of the common situation where two entities (say, **A** and **B**) are competing for limited resources. There are two possible outcomes. On the one hand, if **A** initially gains more of the resources than **B**, it will be able to compete more strongly than **B**, further increasing its share of the resources and so its competitive advantage. The reinforcing feedback loops work together to amplify this disparity, eventually driving **A** to success and **B** to failure. On the other hand, if **B** initially gains a greater share of the resources, the feedback loops will drive **B** to success and **A** to failure. The rich get richer, and the poor get poorer.

In the version of the archetype shown in Figure 9 the competitors are two approaches to urban design—one that promotes active and public transport, and one that assumes that everyone will have access to a car. In the case where car dependence increases over time the dynamical story told by Figure 9 reads as follows:

Loop R1—As the level of **Individual dependence on private motor vehicles** increases, it reduces the pressure on urban designers to promote facilities for active and public transport. This pressure reduction leads to cities with fewer such facilities, further increasing the need for individuals to depend on cars—and so on around the loop. Historically this reinforcing feedback process has driven car dependence upwards over time.

Loop R2—As the level of **Individual dependence on private motor vehicles** increases, so the tendency for urban designers to assume that the majority of citizens have access to a car also increases. This assumption leads to cities where access to a car is necessary for every-day operations, further increasing the level of **Individual dependence on private motor vehicles**. As was the case for Loop R1, this reinforcing process has increased car dependence in most cities.

Reinforcing structures like that shown in Figure 9 give rise to the dynamic phenomena of “path dependence” and “lock-in” [45]. Loops R1 and R2 work together to drive city planning in one direction or the other—towards either dominance of cars, or dominance of active and public transport. How a city develops over time is path dependent. As soon as a bias toward one urban form or the other emerges the reinforcing structures take hold and drive development further in that direction, thus locking in the bias. Once that has happened it is difficult to see how to move the city over to the alternative form. It is unlikely that the Figure 6 balancing loops alone will have the necessary power. The city must first be moved to an unstable (tipping point) state, where both urban forms have approximately equal weight. Depending on the approach taken, this task can appear to be extremely difficult (even impossible)—or very easy. It depends on whether or not an effective leverage point can be identified.

Leverage points are places where small efforts (or precisely directed, simple strategies) produce large results [5,14]. Their identification, in systems as complex as cities, requires a feel for the dynamics of the system. That this can be done in practice has been demonstrated in the downtown area of Portland, Oregon. According to Marshall [46]:

To have a cohesive downtown, or really even older neighbourhoods, a city has to have a cohesive system of mass transit, and it has to make it dominant, or at least close to dominant. Urbanism is a result of pressure. It’s about putting people, activities, and movement in a confined space. Only mass transit has the ability to raise the pressure to enough people per square inch; cars release pressure as surely as puncturing a hole in a tire….

Portland increased its urban pressure by prohibiting, in the mid-1970s, the construction of more parking spaces. This was a master stroke, a strategy opposite that of most other places. Other cities perversely *required* the construction of parking spaces. If you built an office building, you were required to build an even larger parking box beside it to house the cars…. And even without laws, office builders would usually construct parking so their customers or workers would have an easy way of getting to and from their offices or stores.

By prohibiting the construction of parking, Portland managed to reverse this dynamic. It was a pressure builder. Any new businesses or stores or homes would have to make do with the parking that was there. This pushed people onto the buses and eventually onto the light rail line. Of course, without a growth boundary businesses and stores might have just left downtown altogether. But with the growth boundary, it was not as easy to move outward, even though a significant chunk of open land remained within it. The boundary kept the pattern of development still relatively constrained.

Marshall goes on to explain how the Portland parking cap arose. The United States Environmental Protection Agency required the city to find a way to deal with its poor air quality and level of automobile emissions. Thus, their introduction of the parking cap was a result of concerns of the type that drive the balancing co-effects loops of Figure 6. In terms of the Success to the Successful structure of Figure 9, it reduced the extent to which Portland designers assumed that travellers to the downtown area would use cars—indeed, they moved to an urban design that strongly discouraged such travel. This action reduced the level of individual dependence on cars and, in turn, led to an increase in the extent to which their urban design promoted active and public transport. The R1-R2 structure then worked to lock in the low-car-dependence state.

The area devoted to parking is clearly a potential leverage point in any car-dominated city. It affects the cityscape in many ways [47] and has a powerful effect on individuals’ travel decisions and on the attention that planners pay to active and public transport. As demonstrated in Portland, a parking cap used in conjunction with a city growth limit is a relatively simple strategy that can drive large changes. It is unfortunate that commercial pressures, exerted by the operators of parking facilities, have led to a relaxation of the Portland parking cap in recent years [48]. In Australia, the City of Melbourne’s Transport Strategy 2012-30 proposes a similar measure. It raises the possibility of capping the number of long-term commuter car spaces available in new office developments. To complement this, there are plans to expand the city’s walking and bicycle network [49].

The above discussion has centred on feedback loops. Meadows [5] considers changes to the strength of feedback loops as moderately effective leverage points. City planners’ ability to recognise the existence and practical power of such leverage points can be enhanced if they can take a systems approach. This points to another, potentially much more powerful, leverage point that is near the top of the Meadows scale—namely, the paradigms, or mind-sets, from which urban systems arise. We suggest that the development of a set of simple conceptual models, and discussion of their behaviour and potential interactions, provides a practical way to push on this leverage point. It can help policy makers and managers to see how to apply system dynamics principles, in a disciplined way, to capture their experience-based intuitions about system behaviour. Such an approach empowers its practitioners by increasing their ability to learn from experience in complex situations, and to apply their accumulated knowledge in the construction of adaptive plans.

All adaptation decisions involve an attempt to predict the effectiveness of alternative actions. But, prediction is not possible without some kind of dynamical model of how things might change as time passes. A group’s models-in-use will range from informal mental models, held by individual group members, to formal computer models developed by professional modellers and made available to all members of the group. Taken together, these causal models will constitute the group’s understanding of the dynamics of their system-of-interest. Some of this understanding will be shared, some of it private. The shared understanding is central to group’s adaptive capacity. It will be a key component of their systemic understanding and so will enhance their ability to separate adaptive actions from maladaptive actions—and to identify effective leverage points and decide which way to push them.

The main aim of modelling is not a model, but improved understanding. There is ample evidence that the construction of conceptual models, such as those presented in this paper, is a practical way for a group to expand their understanding of the range of things that can happen when humans take action in complex systems. Such models become the basis for a powerful metaphorical understanding that can be extended to a wide range of systems [19,50]. In addition, if the conceptual modelling process described here is extended to the collaborative development and simulation of simple stock-and-flow models, then the group can develop even deeper levels of understanding [5,13,14,50,51,52].

While there is a growing awareness of the need for a systems approach to adaptation, at present few communities are capable of taking such an approach. Indeed, as Sterman [53] has demonstrated, there is a widespread lack of understanding of the dynamics of even very simple stock-and-flow structures. This is a serious situation, but once it is recognised one thing becomes obvious—with such a low base-line even a small increase in a community’s understanding of system dynamics can result in a significant increase in their adaptive capacity.

## 7. Conclusions

In this paper we have described a practical, three-step approach to the development of simple conceptual models that can be used to enhance a group’s understanding of the dynamics of complex managed systems. This approach involves moving from (a) an abstract system template, that establishes a focus on a particular high-level aspect of the behaviour of human-environment systems, through (b) the selection of a problem space that is tailored to match the type of management problem of concern, to (c) a tightly defined conceptual model of a specific sub-system.

Conceptual models with simple feedback structures, like those discussed in this paper, can be developed in a reasonable amount of time using step-by-step, system-dynamics-based approaches like those followed in a CCM endeavour. If the members of an adaptive-planning group are to work together on the development of causal models, then they must aim for models that are simple enough to be built relatively quickly and that have understandable dynamics. A small set of such models can be used to express hypotheses that capture key aspects of the dynamics of the group’s system-of-interest. Significant steps toward improved understanding can be made without connecting these models together to build more complicated representations. The sub-systems described in Figure 6, Figure 8 and Figure 9 have a stock in common, **Individual dependence on private motor vehicles**, and so it is not difficult to imagine the interactions that might take place between them. Discussion of such systemic interactions can greatly increase a group’s understanding of the behaviour of their system-of-interest, and of system dynamics in general.

Clearly, an understanding of the dynamic hypotheses presented in Figure 6, Figure 8 and Figure 9 does not constitute a full understanding of urban climate-health dynamics. Our argument is that the *process* of constructing and discussing such conceptual models, following procedures like those described here, is likely to help a group develop an improved systemic understanding and greater adaptive capacity.

Furthermore, conceptual models can provide an effective starting point for stock-and-flow modelling. Working stock-and-flow models can be simulated to explore the implications of various policies and adaptive strategies. Sensitivity tests can be run to estimate the relative importance of various state-change processes, and to study cross-scale interactions and the way that accumulation gives rise to policy inertia. Simulation experiments can help a group to test their intuitions concerning potential leverage points for adaptation, and to determine which way to push them.

Practical systems-thinking approaches, like that outlined here, can play an important role in the development of urban policy. An urban community’s capacity to maintain high levels of public health and well-being, in the face of climate change, will ultimately depend on their ability to appreciate that a range of maladaptive outcomes can flow from a given management decision. And that capacity, in turn, will depend on the extent to which the community learns to see with system dynamics eyes.
